# RAFT-mediated Pickering emulsion polymerization with cellulose nanocrystals grafted with random copolymer as stabilizer†

**DOI:** 10.1039/c8ra03816c

**Published:** 2018-08-13

**Authors:** Liangjiu Bai, Xinyan Jiang, Beifang Liu, Wenxiang Wang, Hou Chen, Zhongxin Xue, Yuzhong Niu, Huawei Yang, Donglei Wei

**Affiliations:** School of Chemistry and Materials Science, Key Laboratory of High Performance and Functional Polymer in the Universities of Shandong Province, Ludong University Yantai 264025 China wenxiang-tianshi@163.com ldupolymchen@163.com +86-535-6669070 +86-535-6697933; Collaborative Innovation Center of Shandong Province for High Performance Fibers and Their Composites, Ludong University Yantai 264025 China

## Abstract

The synthesis of a RAFT-mediated Pickering emulsion was firstly achieved by using cellulose nanocrystals (CNCs) grafted with a random copolymer as the stabilizer. Firstly, poly(acrylonitrile-*r*-butyl acrylate) (poly(AN-*r-n*BA)) was synthesized by Cu(0)-mediated CRP, which was further modified *via* a click chemistry strategy to obtain poly(ethylene tetrazole-*r*-butyl acrylate) (poly(VT-*r-n*BA)). Then, poly(VT-*r-n*BA) was grafted onto the CNCs through a Mitsunobu reaction to obtain poly(VT-*r-n*BA)-*g*-CNCs. Stabilized by poly(VT-*r-n*BA)-*g*-CNCs, an O/W RAFT-mediated Pickering emulsion was formed for the preparation of well-controlled poly(methyl methacrylate) (PMMA) particles with water-soluble potassium persulfate (KPS) as an initiator and oil-soluble 4-cyanopentanoic acid dithiobenzoate (CPADB) as a chain transfer agent. Rheological analysis suggested that the prepared Pickering emulsion possessed good stability under the influences of changes in strain, time, frequency and temperature. Furthermore, the recycling and further utilization of the poly(VT-*r-n*BA)-*g*-CNCs could be simply realized through centrifugal separation.

## Abstract 2

A RAFT-mediated Pickering emulsion with cellulose nanocrystals grafted with a random copolymer was used for the preparation of poly(methyl methacrylate) particles..

## Graphical abstract

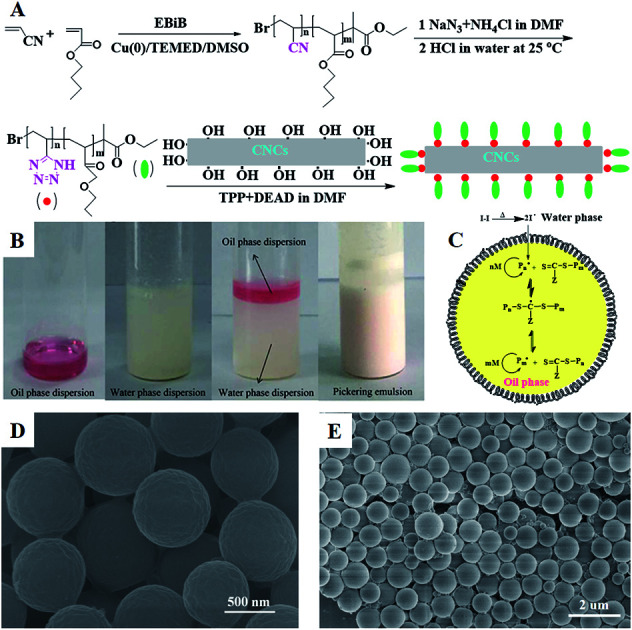

## Introduction

Surface modification plays a significant role in the “top-down” construction of functional materials.^1^ Among surface modifiers, random copolymers have recently attracted much attention due to their convenient design and high functional group density,^2^ and are widely employed in various fields such as controlled drug delivery,^3^ membrane materials,^4^ reinforcement,^5^ absorption materials,^6^ thermoresponsive materials^7^ and antibacterial materials.^5^ Attractive performances have also been achieved in the application of their mechanical properties, bio-absorbability, biodegradability, cytocompatibility and optical functions.^8^ More recently, the functionalization of cellulose with random copolymers has been widely applied in the bio-related, agroindustrial and chemical industries.^9^ Cellulose can be easily recycled for further utilization, and can be separated from products.^10^ Therefore, the use of cellulose-grafted copolymers makes it possible to achieve the dual advantages of cellulose and random copolymers.^11^

It should be noted that random copolymers can be used solely as emulsifiers to stabilize emulsions.^12^ However, it is difficult to provide high performances compared to those of block copolymers.^13^ Furthermore, their solubilities in solvents make the separation and purification of the products more difficult in the post-treatment of emulsion polymerizations. In contrast, for polymerization in a Pickering emulsion, it was reported that the products can be easily isolated and the emulsifier can be readily recycled, respectively. Pickering emulsions are also named particle-stabilized emulsions,^14^ in which particles are absorbed at the phase interface to stabilize the emulsion without additional surface-active agents.^15^ Compared with conventional emulsion polymerizations,^16^ Pickering emulsion polymerization is economically attractive because of the enhanced stability and less of a requirement for stabilizers.^17^ Therefore, Pickering emulsion polymerization exhibits great potential in industrial application in cosmetics, pharmaceuticals, food products and coatings.^18^

Faia Laredj-Bourezg and co-workers investigated a surfactant-free biodegradable and biocompatible O/W Pickering emulsion using block copolymer nanoparticles as a stabilizer for controlled topical drug delivery.^19^ Hou and co-workers reported that P4VP-*b*-PS and PS-*b*-P4VP grafted onto halloysite nanotubes were used to stabilize a Pickering emulsion.^20^ In particular, environmentally friendly cellulose nanocrystals (CNCs) can also act as stabilizers in Pickering emulsion systems,^21^ which are sufficiently insensitive to the material source and charge density due to the super-hydrophilic properties of CNCs.^22^ Additionally, CNCs as typical nano-reinforcers, have reinforcement effects due to the surface-grafted functional polymers that improve their compatibilities with substrates. For example, polymers such as thermo-responsive poly(*N*-isopropylacrylamide),^23^ poly(lactic acid),^24^ and a thermo-sensitive and pH-sensitive poly(*N*-isopropylacrylamide-*co*-acrylic acid) copolymer^25^ were successfully grafted by controlled radical polymerization or ring-opening polymerization, which can improve the compatibility of CNCs to strengthen the substrate. Poly(butylene succinate) (PBS)/polylactic acid (PLA) blends modified with dicumyl peroxide (DCP) were reinforced by PBS-*g*-CNCs^26^ by *in situ* polymerization. In order to improve the application range of CNCs in Pickering emulsion polymerizations, CNC-grafted polymers for improving the hydrophobicity of CNCs and compatibility with polymers have also been reported. Dual responsive Pickering emulsifiers based on CNCs were synthesized by grafting PDMAEMA onto CNCs *via* a free radical polymerization process.^27^ Orlando J. Rojas *et al.*^18*b*^ have achieved efficient oil-in-water emulsions by using CNCs grafted with thermo-responsive polymers (PNIPAM) as stabilizers. However, reactions such as surface-initiated CRP or free radical polymerizations are limited by the reaction efficiency of a polymer’s end groups. Block copolymers on CNCs can also increase the interfacial stability of Pickering emulsion polymerizations. These synthesis and graft processes are also related to end group activity and surface initiation efficiency.

However, there are few reports on the surface grafting of random copolymers onto CNCs. Alain Dufresne *et al.*^28^ successfully extended polystyrene by modifying CNCs with poly[(styrene)-*co*-(2-ethylhexylacrylate)]. AN and *n*BA-type random copolymers as typical functional polymers have been widely reported, and the synthesis and controlled processes are similar to the homo-polymerization process. As a large number of tetrazole groups in poly(VT-*r-n*BA) provide more reaction sites, the modification of the CNCs is more efficient, and the raw materials are relatively inexpensive, as poly(AN-*r-n*BA) can be converted to functional tetrazole groups by sodium azide and can be grafted onto the surface of CNCs by the Mitsunobu reaction. The hydrophobic functional groups on the surface (polymer backbone and *n*BA side chains) and the hydrophilic cellulose backbone play key roles in stabilizing Pickering emulsions. In this work, novel copolymer-grafted CNCs, named poly(acrylonitrile-*r*-butyl acrylate)-*g*-cellulose nanocrystals (poly(VT-*r-n*BA)-*g*-CNCs), were simply and efficiently synthesized *via* the combination of a Cu(0)-mediated CRP process, click chemistry and a Mitsunobu reaction. Afterwards, a living RAFT-mediated O/W Pickering emulsion, using the obtained poly(VT-*r-n*BA)-*g*-CNCs as the sole stabilizer, was ingeniously designed for the preparation of well-controlled poly(methyl methacrylate) (PMMA) particles. The rheological behaviour of the Pickering emulsion was measured under varying strain, frequency, time and temperature conditions. To verify the recoverability of the obtained cellulosic grafted copolymer, the polymerization behaviour of the Pickering emulsion for PMMA production, employing recycled poly(VT-*r-n*BA)-*g*-CNCs as a stabilizer, was studied. The utilization of the CNCs grafted with random copolymers could not only improve the separation and recovery of the modified CNC nanoparticles from the Pickering emulsion polymerizations, but could also improve the dispersibility of the modified CNCs and the stability of the emulsion.

## Experimental section

### Materials

Acrylonitrile (AN), *n*-butyl acrylate (*n*BA) and methyl methacrylate (MMA) were purchased from Tianjin FuChen Chemical Reagents Factory and the inhibitors of the reagents were removed by neutral alumina column before use. Ethyl 2-bromoisobutyrate (EBiB), *N*,*N*,*N*′,*N*′-tetramethylethylene (TMEDA), 4-cyano-4-(phenylcarbonothioylthio)pentanoic acid (CPADB) and diethyl azodicarboxylate (DEAD) were obtained from Zhengzhou Alfa Chemical Company (China). Cu(0) powder, sodium azide (NaN_3_), ammonium chloride (NH_4_Cl), microcrystalline cellulose (MCC), triphenylphosphine (TPP) and potassium persulfate (KPS) were purchased from Tianjin Damao Chemical Reagents Factory (China). Sulfuric acid (H_2_SO_4_), *N*,*N*-dimethylformamide (DMF) and dimethyl sulfoxide (DMSO) were obtained from Laiyang Fine Chemical Factory (China). Deionized water that was purified through a UPH-I-40L super-pure water machine was used in all Pickering emulsions.

### Preparation of poly(acrylonitrile-*r*-butyl acrylate) (poly(AN-*r-n*BA))

Poly(AN-*r-n*BA) was synthesized by a Cu(0)-mediated CRP with the reaction conditions of [monomer] : [EBiB] : [Cu(0)] : [TMEDA] = 200 : 1 : 1 : 2, [AN] : [*n*BA] = 1 : 1. Cu(0) (9.7 mg, 0.153 mmol) as a catalyst, AN (1 mL, 0.0153 mol) as a monomer, BA (2.2 mL, 0.0153 mol) as a monomer, EBiB (0.022 mL, 0.153 mmol) as an initiator, TMEDA (0.045 mL, 0.306 mmol) as a ligand and DMSO (2 mL) as a solvent were added to a 7 mL ampoule in order. Then, the ampoule was placed in ice water and the contents were deoxygenated with nitrogen for 10 min. The ampoule was then sealed by an ampoule sealer and put in an oil-bath at 70 °C for a predetermined time. Afterwards, the reaction mixture was fully dissolved in DMSO and then completely precipitated with methanol. Finally, the polymer was dried by a vacuum freeze drier and the conversion was calculated *via* the weighing method.

### Preparation of poly(vinyltetrazole-*r*-butyl acrylate) (poly(VT-*r-n*BA)) *via* click chemistry

Poly(VT-*r-n*BA) was synthesized by click chemistry of poly(AN-*r*-nBA) and sodium azide. In a 7 mL ampoule, poly(AN-*r-n*BA) (0.150 g), NaN_3_ (0.054 g) and NH_4_Cl (0.044 g) were sufficiently dissolved in 4 mL of DMF. Then, the mixture was put in an oil-bath and reacted at 120 °C for 12 h. After the reaction, the mixture solution was cooled to ambient temperature and then slowly added to 1 mol L^−1^ HCl for complete precipitation. The product was then filtered, washed with excess aqueous HCl solution and dried by freeze-drying.

### Preparation of the cellulosic graft copolymer poly(VT-*r-n*BA)-*g*-CNCs *via* Mitsunobu reaction

Cellulose nanocrystals (CNCs) were prepared by the sulfuric acid hydrolysis of microcrystalline cellulose (MCC).^29^ Then, poly(VT-*r-n*BA)-*g*-CNCs were synthesized by the Mitsunobu reaction of poly(VT-*r-n*BA) and CNCs. In a 100 mL round-bottomed flask, poly(VT-*r-n*BA) (100 mg) and CNCs (100 mg) were dissolved and dispersed in 15 mL of DMF. Then, TPP (0.360 mg) and DEAD (260 μL) were added under nitrogen in an ice-water bath. Thereafter, the flask was put in a 25 °C oil-bath with stirring for 22 h. After dialysis and lyophilization, the nanocomposite was obtained.

### Preparation of the Pickering emulsion

The Pickering emulsion stabilized by CNCs-grafted copolymer poly(VT-*r-n*BA)-*g*-CNCs was produced by RAFT polymerization of PMMA. In a 7 mL ampoule, poly(VT-*r-n*BA)-*g*-CNCs were evenly dispersed in 3 mL deionized water by ultrasonic agitation. Then 0.0031 g of KPS was added to the poly(VT-*r-n*BA)-*g*-CNCs dispersion as the water phase. Also, the RAFT chain transfer agent CPADB (0.0026 g) was dissolved in 0.5 mL of MMA as the oil phase. Subsequently, the oil phase was shifted dropwise to the water phase with stirring. Then the resulting solution was ultrasonicated and deoxygenated with high purity nitrogen under ice-water conditions for 10 min. Soon after, the ampoule was sealed and placed in a 60 °C oil-bath. Finally, the polymer was obtained by centrifugation, dialysis, freeze-drying and gravimetric filtration. The poly(VT-*r-n*BA)-*g*-CNCs stabilized the Pickering emulsion and were reutilized as a stabilizer of Pickering emulsions.

### Characterization

Energy dispersive X-ray spectroscopy (EDS) and scanning electron microscopy (SEM) images were obtained using a JEOL JSM-5610 LV SEM. Rheological properties were measured using a TA instruments-Waters LLC DHR-3 rheometer. FTIR spectra were recorded using a Nicolet Magna 550 Series II FTIR spectrophotometer. The ^1^H NMR and ^13^C NMR spectra were obtained using an INOVA-400 MHz NMR instrument. Dynamic light scattering (DLS) and zeta potentials (Zeta) were determined using a Malvern Nano-ZS90. The number-average molecular weight (*M*_n_) and the molecular weight distribution (*M*_w_/*M*_n_) of the polymers were determined with a Waters 1515-2414 GPC using a column (7.8 × 300 mm and 5 μm beads size) with chromatographically pure DMF as the eluent. X-ray photoelectron spectroscopy (XPS) measurements were recorded with an ESCALAB Xi^+^ (Thermo Scientific, United States) photoelectron spectrometer.

## Results and discussion

### Preparation and characterization of poly(VT-*r-n*BA)-*g*-CNCs

**Fig. 1:**
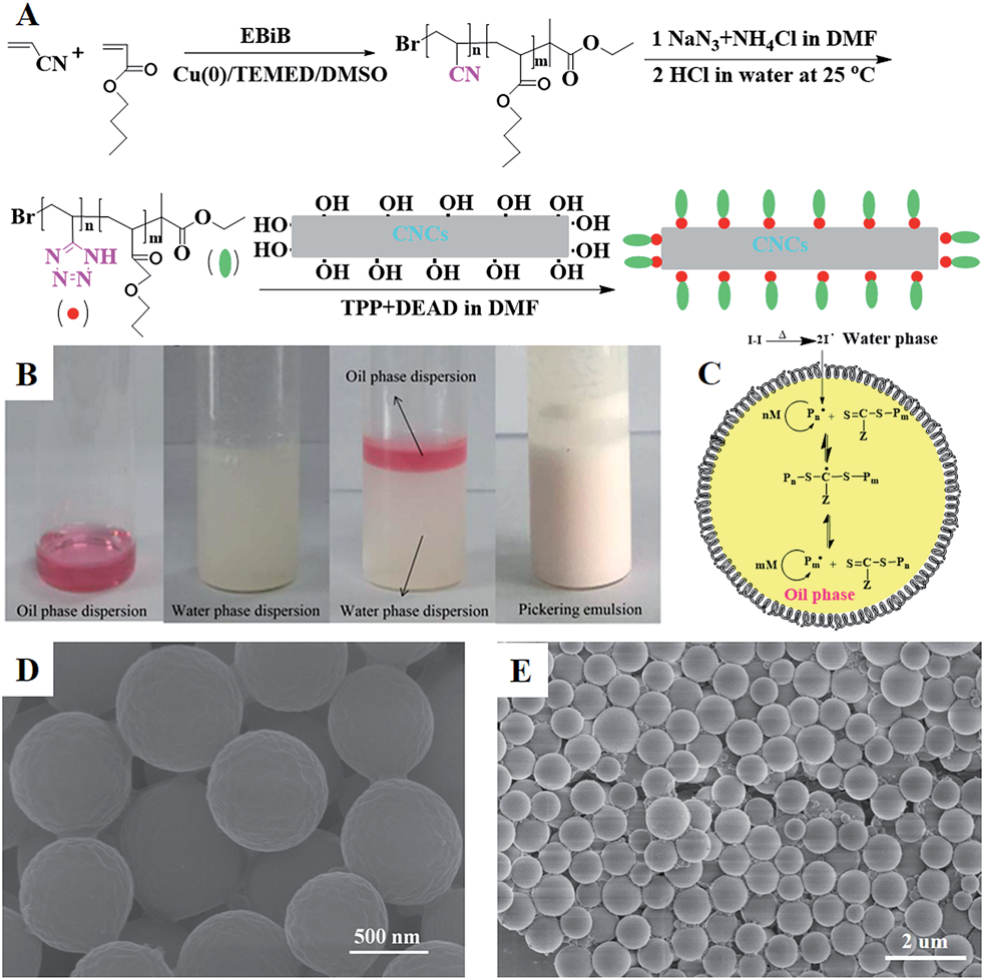
(A) The preparation process of modified poly(AN-*r-n*BA) with CNCs; (B) photographs of (1) the oil phase dispersion, (2) and the water phase dispersion, (3) before and (4) after Pickering emulsion polymerization stabilized by poly(VT-*r-n*BA)-*g*-CNCs; (C) synthesis mechanism of the RAFT-mediated Pickering emulsion polymerization of MMA stabilized by poly(VT-*r-n*BA)-*g*-CNCs; (D), (E) SEM images of the PMMA prepared *via* the Pickering emulsion polymerization.

**Fig. 2:**
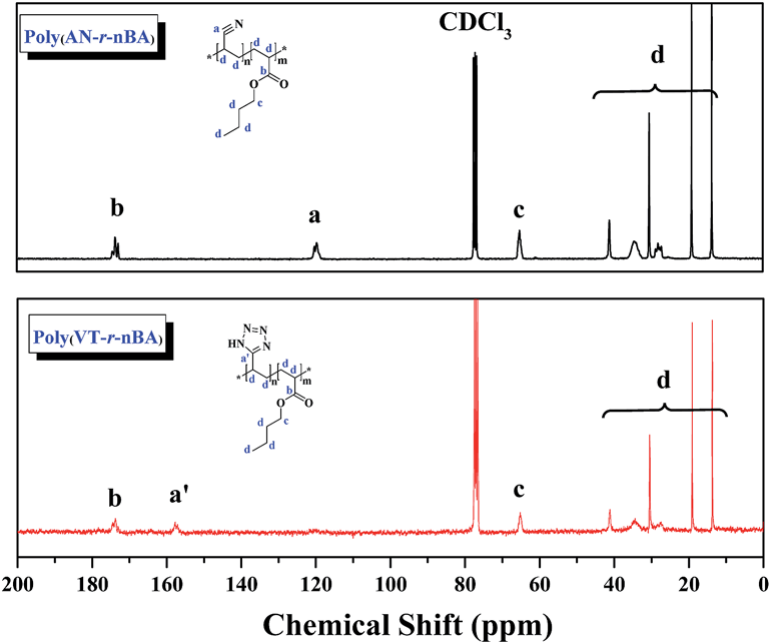
The ^13^C NMR spectra of poly(AN-*r-n*BA) and poly(VT-*r-n*BA) with CDCl_3_ as the solvent.

In this work, well-defined poly(VT-*r-n*BA)-*g*-CNCs were successfully prepared according to the route shown in Fig. 1A. Table S1† summarizes the EDS data of the CNCs, poly(AN-*r*-BA), poly(VT-*r*-BA) and poly(VT-*r-n*BA)-*g*-CNCs, and the corresponding EDS pictures are provided in Fig. S2.† Firstly, poly(AN-*r-n*BA) was synthesized by Cu(0)-mediated CRP with the reaction conditions of [AN] : [*n*BA] : [EBiB] : [Cu(0)] : [TMEDA] = 100 : 100 : 1 : 1 : 2. The molar ratio of monomers that make up the copolymer can be calculated by the mass ratio, based on the elements of nitrogen and oxygen derived from the monomers of AN and *n*BA, respectively. The mass content of the elements in the copolymer obtained by EDS can be converted to the [N]/[O] molar ratio. The contents of C, O and N elements in poly(AN-*r*-BA) were determined to be 69.70%, 19.10% and 11.20% respectively (Table S1†), which indicated that the amount of AN_P(AN-*co*-BA)_ : BA_P(AN-*co*-BA)_ was 1.34 : 1.00 with the molar ratio of [N]/[O]. Then, as reported in the literature,^30^ the synthesis of poly(VT-*r-n*BA) was achieved by a click reaction between the nitrile group of poly(AN-*r-n*BA) and sodium azide. Similarly, the VT/BA molar ratio in poly(VT-*r*-BA) was calculated to be 1.32 : 1.00 with the EDS results of C, O and N elements. Meanwhile, CNCs were prepared by the sulphuric acid hydrolysis method, and gave the TEM image shown in Fig. S1.† Finally, the poly(VT-*r-n*BA)-*g*-CNCs were synthesized by a Mitsunobu reaction between poly(VT-*r-n*BA) and the CNCs. The grafting percent was approximately 78.3%, as measured by the C, O and N elemental composition of the CNCs, poly(VT-*r*-BA) and poly(VT-*r-n*BA)-*g*-CNCs. In order to further prove the structures of the polymers, Fig. 2 shows the ^13^C NMR spectra of poly(AN-*r-n*BA) and poly(VT-*r-n*BA), respectively. It can be seen that the peaks at 120 ppm, 173 ppm and 64 ppm represent the nitrile groups and the ester groups and the alkyl group connected to an oxygen atom (C(O)O–CH_2_) in the butyl acrylate unit, respectively. When the poly(AN-*r-n*BA) was transformed into poly(VT-*r-n*BA), the peak of the nitrile-based carbon atom was shifted to 157 ppm, which was consistent with the literature^30*a*^ (the azole ring at 157 ppm). Therefore, the functional poly(VT-*r-n*BA) was successfully synthesized. The FT-IR spectra of poly(AN-*r*-BA), poly(VT-*r*-BA), CNCs and poly(VT-*r*-BA)-*g*-CNCs are exhibited in Fig. S3.† These results indicate the successful synthesis of poly(VT-*r-n*BA)-*g*-CNCs using poly(AN-*r-n*BA) and CNCs as raw materials.

**Fig. 3:**
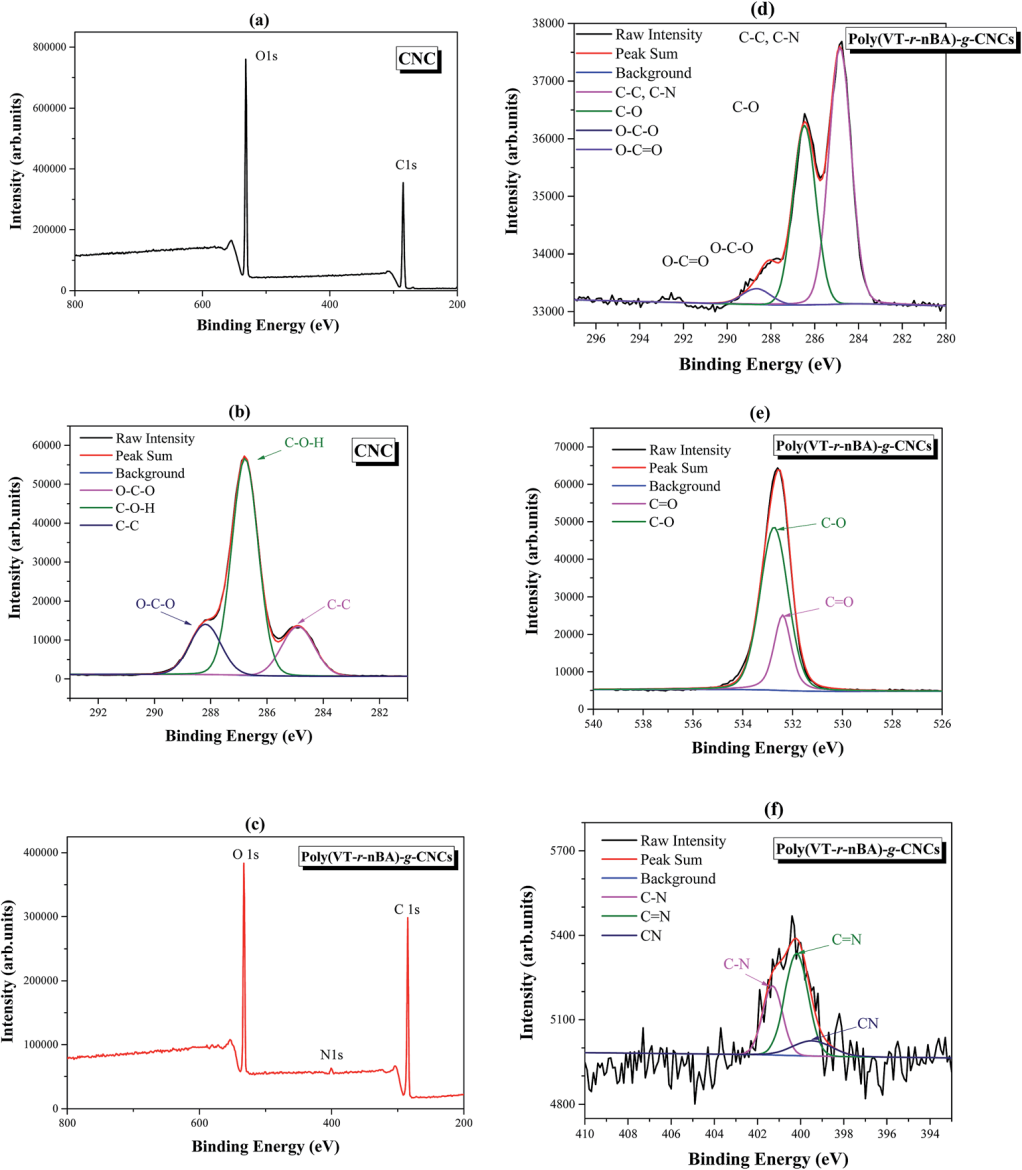
The XPS spectra of the CNCs and poly(VT-*r-n*BA)-*g*-CNCs, (a) wide scan XPS for the CNCs; (b) high resolution spectra for C 1s of the CNCs; (c) wide scan XPS for poly(VT-*r-n*BA)-*g*-CNCs; (d) high resolution spectra for C 1s of poly(VT-*r-n*BA)-*g*-CNCs; (e) high resolution spectra for O 1s of poly(VT-*r-n*BA)-*g*-CNCs; (f) high resolution spectra for N 1s of poly(VT-*r-n*BA)-*g*-CNCs.

As an important analytical method for surface modification, X-ray photoelectron spectroscopy (XPS) can be used to fully analyze the surface element characteristics of materials. As shown in Fig. 3(a), the peaks at 284.8 eV, and 531.6 eV in wide scan XPS are attributed to the C 1s and O 1s peaks of the CNCs. The C 1s spectra of the CNCs in Fig. 3(b) can be fitted to a curve with three peak components, which are attributed to C–O–H (286.8 eV), O–C–O (288.4 eV) and C–C (284.8 eV), respectively. The results are in good agreement with those in the literature,^31^ which proves the successful preparation of the CNCs. In Fig. 3(c), the peaks of wide scan XPS at 284.8 eV, 400.5 eV, and 532.8 eV are attributed to C 1s, N 1s and O 2p in poly(VT-*r-n*BA)-*g*-CNCs, respectively. Compared with the wide scan XPS of the CNCs, the N 1s peak in poly(VT-*r-n*BA)-*g*-CNCs indicated that the surfaces were successfully grafted with the random polymer P4VT–P*n*BA. From Fig. 3(d), the C 1s spectra of the poly(VT-*r-n*BA)-*g*-CNCs in Fig. 3(b) can be fitted to a curve with four peak components, which are attributed to C–C or C–N (284.8 eV), C–O (286.5 eV), O–C–O (288.0 eV), and O–C=O (288.6 eV), respectively. The characteristic peaks of the CNCs (O–C–O) and poly(VT-*r-n*BA) (O–C=O) proved that the CNCs were successfully modified. Fig. 3(e) is the O 1s spectrum of poly(VT-*r-n*BA)-*g*-CNCs with the O 1s peaks at 532.7 eV and 532.3 eV attributed to C–O, and C=O, respectively. Due to the presence of C–O bonds in the cellulose nanocrystals, the C–O peak area is larger than that of C=O. Fig. 3(f) is the N 1s spectrum of poly(VT-*r-n*BA)-*g*-CNCs and the N 1s peaks at 401.3 eV, 400.2 eV and 399.5 eV are assigned as the peaks for C–N, C=N and C≡N, respectively. Therefore, the results of the XPS fully demonstrate that poly(VT-*r-n*BA) was grafted to the surface of the CNCs.

### Preparation of PMMA by a RAFT-mediated Pickering emulsion with poly(VT-*r-n*BA)-*g*-CNCs as a stabilizer

Using the successfully synthesized poly(VT-*r-n*BA)-*g*-CNCs as a stabilizer, a stabilized and homogeneous Pickering emulsion was prepared (Fig. 1(B)) without any additional surfactants. CPADB was dispersed in MMA to form the oil phase (Fig. 1B_1_), and KPS and poly(VT-*r-n*BA)-*g*-CNCs were dispersed in deionized water to form the water phase (Fig. 1B_2_), respectively. Then, PMMA beads were prepared by oil-in-water RAFT-mediated Pickering emulsion polymerization (Fig. 1B_4_) with MMA as a monomer, water-soluble KPS as an initiator and oil-soluble CPADB as a chain transfer agent under the following conditions: [MMA]_0_ : [KPS]_0_ : [CPADB]_0_ = 400 : 1 : 1, H_2_O : MMA (v/v) = 6 : 1. The proposed mechanism for the RAFT-mediated Pickering emulsion formation is presented in Fig. 1C. As shown in Fig. 1D and E, the PMMA particles prepared *via* Pickering emulsion polymerization were spheres with homogeneous morphology.

**Fig. 4:**
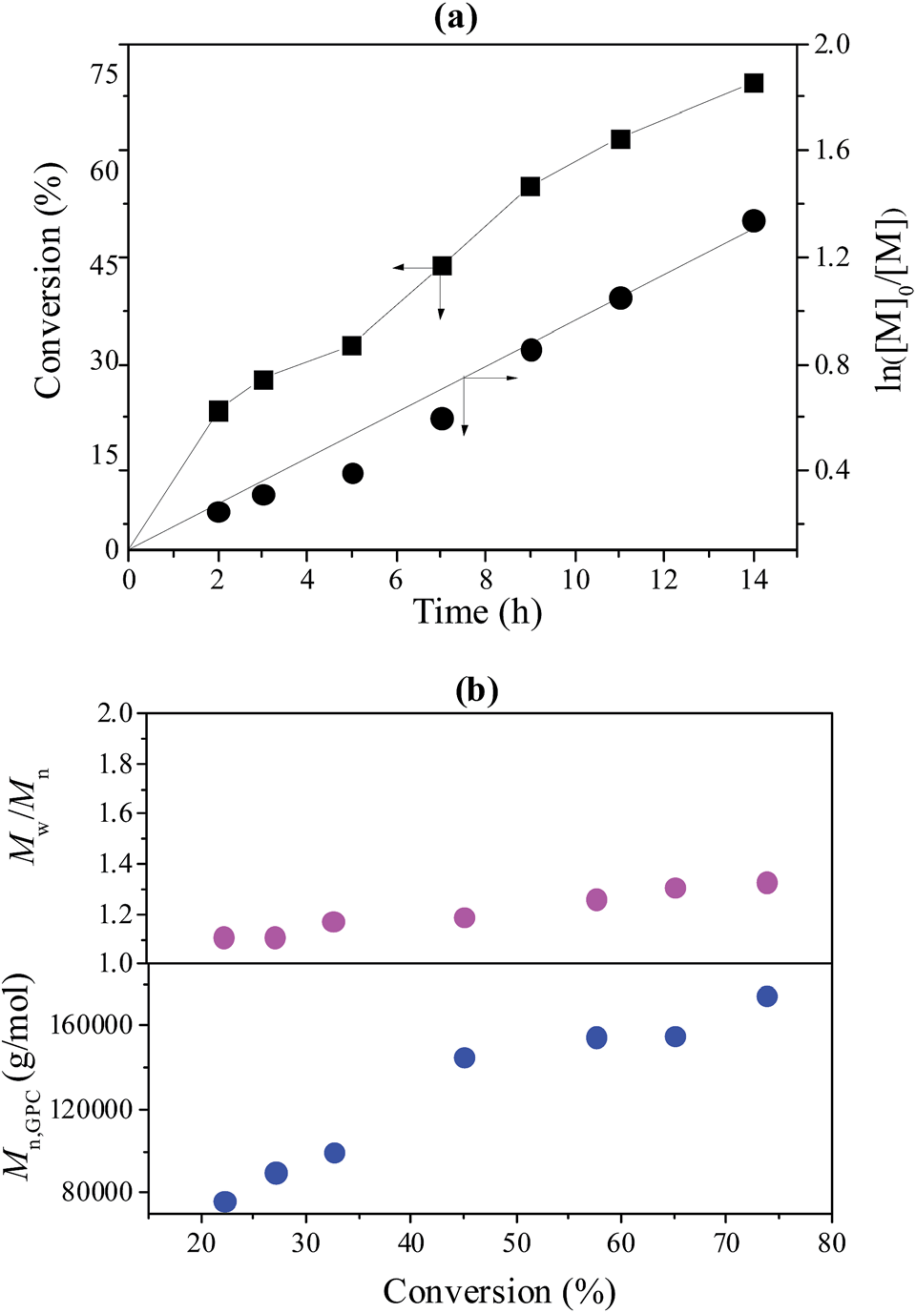
(a) The kinetic plot of PMMA prepared with a RAFT-mediated Pickering emulsion with poly(AN-*r-n*BA)-*g*-CNCs as a stabilizer at 60 °C, [MMA]_0_ : [KPS]_0_ : [CPADB]_0_ = 400 : 1 : 1, H_2_O : MMA (v/v) = 6 : 1; (b) the *M*_n_ and *M*_w_/*M*_n_ of PMMA *versus* monomer conversion.

**Fig. 5:**
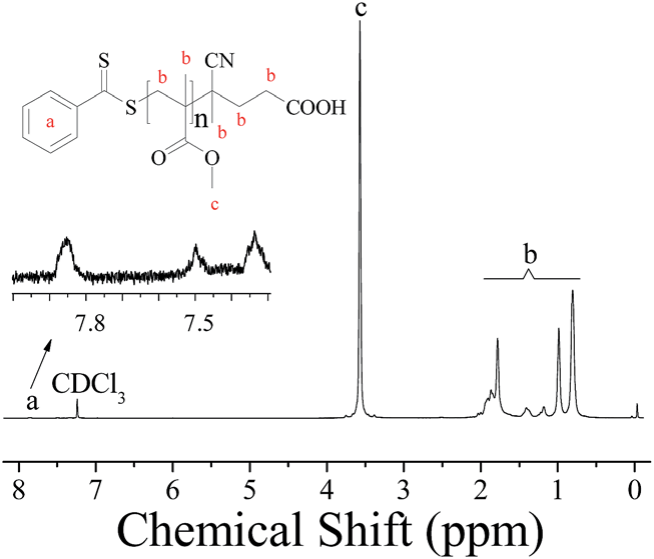
^1^H NMR spectrum of PMMA prepared by RAFT-mediated Pickering emulsion stabilized by poly(VT-*r-n*BA)-*g*-CNCs with CDCl_3_ as the solvent and TMS as an internal standard of chemical-shift.

To further investigate the controllability of the polymerization, Fig. 4(a) shows the kinetic plot of the PMMA synthesis with CPADB as a chain transfer agent. As expected, the conversion of the MMA monomer increased with the increase in polymerization time. A linear relationship between ln([*M*]_0_/[*M*]) and reaction time was observed, which indicated that the polymerization process of MMA proceeded with first-order kinetics (ln([*M*]_0_/[*M*]) = *k*^app^_p_[*R*]*t*). Fig. 4(b) exhibits the relationship of the number average molecular weight of PMMA *versus* monomer conversion, and confirms that low polydispersity of PMMA can be achieved. The ^1^H NMR spectrum of PMMA is shown in Fig. 5. These results demonstrate the successful synthesis of PMMA through RAFT-mediated Pickering emulsion polymerization. The resonances at *δ*_0.73–2.36_ (b) and *δ*_3.6_ (c) were assigned to –CH_2_– and –O–CH_3_ protons of the backbone-chain units and the side-chain terminal of PMMA, while the resonances at *δ*_7.3–7.9_ (a) were attributed to –Ph protons of the chain transfer agent CPADB. Therefore, the RAFT-mediated Pickering emulsion polymerization was proven to possess a controlled/“living” character to synthesize PMMA.

### Rheological analysis of Pickering emulsion

**Fig. 6:**
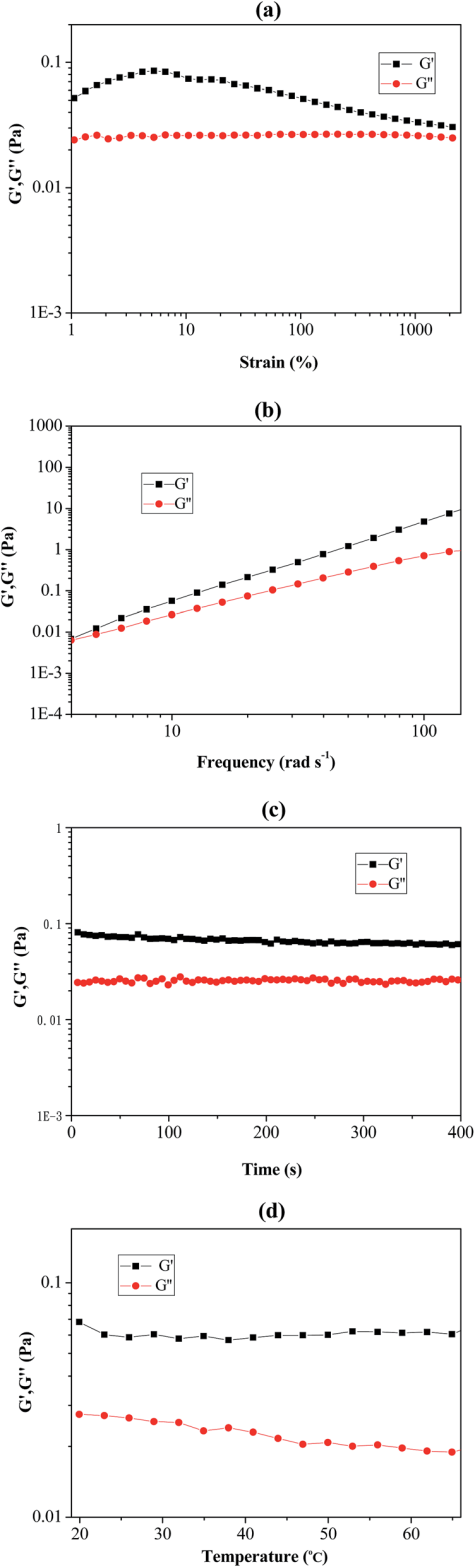
The rheological measurements of PMMA produced by RAFT-mediated Pickering emulsion stabilized by poly(VT-*r-n*BA)-*g*-CNCs. (a) Strain-sweep measurements from 1 to 2500% with 10 rad s^−1^ at room temperature; (b) frequency-sweep measurements from 4 to 140 rad s^−1^ at room temperature; (c) time-sweep measurements from 0 to 400 s with 10 rad s^−1^ at room temperature; (d) temperature-sweep measurements from 20 to 65 °C with 10 rad s^−1^. *G*′: storage modulus; *G*′′: loss modulus.

As shown in Fig. 6(a–d), rheological behaviour of the prepared Pickering emulsion (stabilizer using poly(VT-*r-n*BA)-*g*-CNCs) was measured by using a rheometer. The strain-sweep measurement of the Pickering emulsion is exhibited in Fig. 6(a), and it clearly demonstrated that the loss modulus (*G*′′) remained unchanged under a strain rate ranging from 1% to 1000%. The storage modulus (*G*′) firstly climbed up slightly and then slowly declined with the increase of the strain rate, suggesting that the Pickering emulsion could endure comparatively large deformation. In addition, Fig. 6(b) shows the frequency-sweep measurement results of the Pickering emulsion. *G*′ and *G*′′ increased with increasing frequency, which indicated that the Pickering emulsion remained stable under high-order frequencies. Fig. 6(c) and (d) show the time-sweep and temperature-sweep spectrograms of the Pickering emulsion respectively. It can be seen that *G*′ and *G*′′ did not change with time within 400 s, while *G*′ and *G*′′ presented slight fluctuations under the temperature range from 20 °C to 65 °C. These phenomena sufficiently proved that the prepared Pickering emulsion possessed good stability and viscoelasticity under the influence of changes in strain, time, frequency and temperature.

### Recycling of the poly(VT-*r-n*BA)-*g*-CNCs nanocomposite

**Fig. 7:**
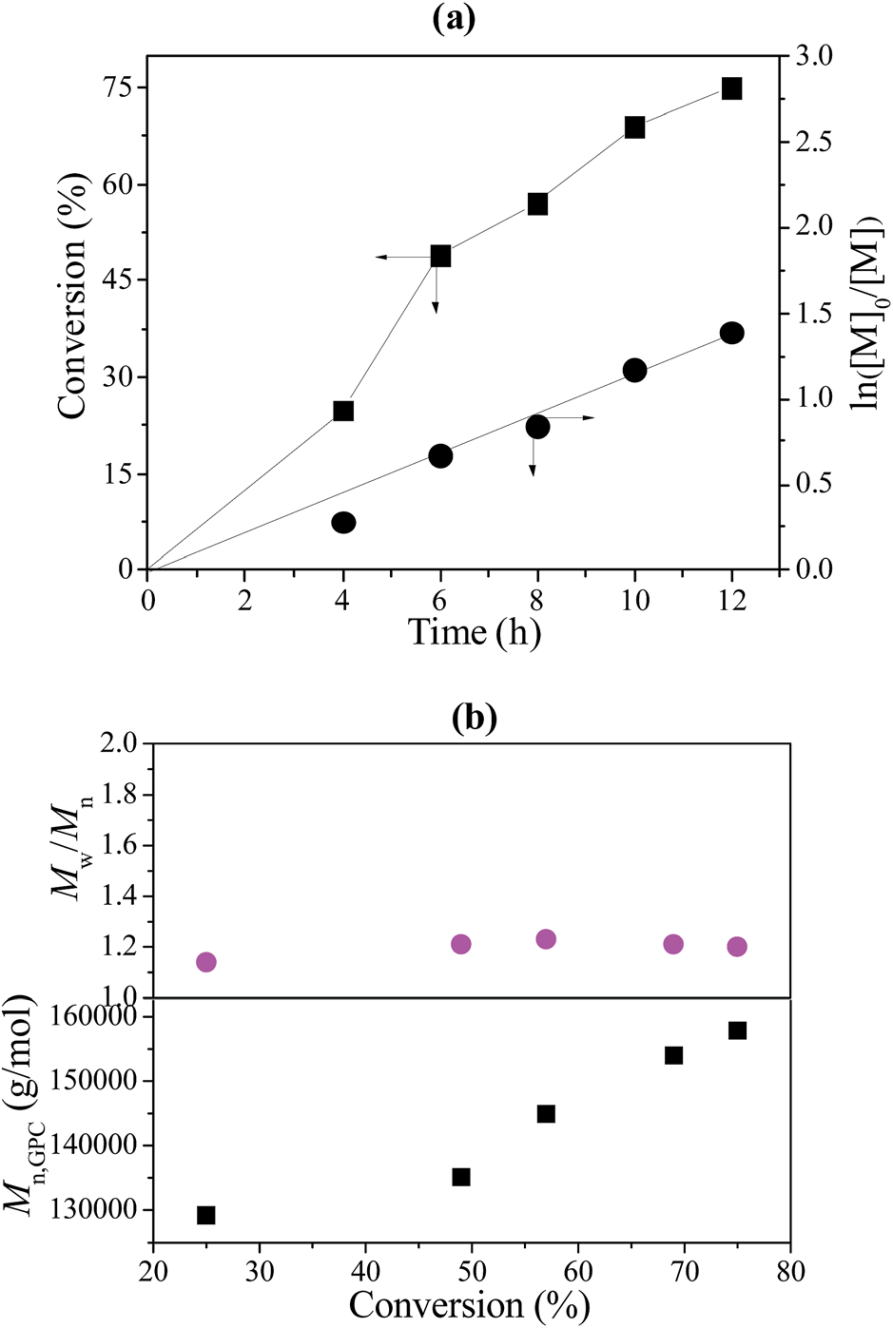
(a) The kinetic plot of PMMA prepared by RAFT-mediated Pickering emulsion with recycled poly(VT-*r-n*BA)-*g*-CNCs as a stabilizer at 60 °C, [MMA]_0_ : [KPS]_0_ : [CPADB]_0_ = 400 : 1 : 1, H_2_O : MMA (v/v) = 6 : 1; (b) the *M*_n_ and *M*_w_/*M*_n_ of PMMA *versus* monomer conversion.

After the reaction, trace THF was added to the polymerization system to dissolve the resulting polymer with sufficient stirring. The mixed polymer solution was centrifuged to separate the poly(VT-*r-n*BA)-*g*-CNCs. The poly(VT-*r-n*BA)-*g*-CNCs were then purified by washing thoroughly with THF, ethanol and water, respectively. At the same time, the polymer solution was poured into a large amount of methanol to obtain the PMMA. Reuse of the poly(VT-*r-n*BA)-*g*-CNCs was carried out for the RAFT-mediated Pickering emulsion polymerization of MMA under the conditions of [MMA]_0_ : [KPS]_0_ : [CPADB]_0_ = 400 : 1 : 1. The Poly(VT-*r-n*BA)-*g*-CNCs stabilized Pickering emulsion was recycled by centrifugal separation. Fig. 7(a) exhibits the relationship of conversion of MMA and ln([*M*]_0_/[*M*]) *versus* reaction time. The results show a positive correlation between conversion of MMA and reaction time. Moreover, ln([*M*]_0_/[*M*]) also had a linear relationship with reaction time. The results fully display the “living”/controlled features of RAFT polymerization. The results of the polymerization kinetics studies indicated that the recycled poly(VT-*r-n*BA)-*g*-CNCs had a *k*^app^_p_ value (*R*_p_ = −d[*M*]/d*t* = *k*_p_[*P*_n_][*M*] = *k*^app^_p_[*M*]) of about 3.1 × 10^−5^ s^−1^, and the reaction rate was faster than that of the initial value (about 2.6 × 10^−5^ s^−1^). The possible reasons are that the emulsification ability of the poly(VT-*r-n*BA)-*g*-CNCs after the recovery was partially reduced after recycling, and the free radicals generated by the initiators were more likely to diffuse into the micelles, resulting in a slightly increased polymerization rate. Also, the *M*_n_ and *M*_w_/*M*_n_ data of PMMA against MMA conversion were plotted in Fig. 7(b). *M*_n_ of PMMA increases with conversion of MMA and their *M*_w_/*M*_n_ remains at a fairly low level. The results indicated that the polymerization of MMA was well-controlled under lower free radical concentrations. Table 1 shows the results of the *M*_n,GPC_ and *M*_w_/*M*_n_ of PMMA prepared using the Pickering emulsion with five cycles of reuse of the poly(VT-*r-n*BA)-*g*-CNCs. It can be observed that the *M*_n,GPC_ values of PMMA have a slightly fluctuating trend with an increasing number of cycles. In addition, the *M*_w_/*M*_n_ values of PMMA have better distribution. This was a good illustration of the successful recycling of the poly(VT-*r-n*BA)-*g*-CNCs.

**Table 1:** Results of the *M*_n,GPC_ and *M*_w_/*M*_n_ of PMMA prepared by Pickering emulsion stabilized with recycled poly(VT-*r-n*BA)-*g*-CNCs

Cycle times	Time (h)	Polymerization conditions	*M*_n,GPC_	*M*_w_/*M*_n_
1	1	[MMA]_0_ : [CPADB]_0_ : [KPS]_0_ = 400 : 1 : 1, H_2_O/MMA (v/v) = 6/1, poly(VT-*r-n*BA)-*g*-CNCs/H_2_O = 4 mg mL^−1^	84 600	1.07
2	1	[MMA]_0_ : [CPADB]_0_ : [KPS]_0_ = 400 : 1 : 1, H_2_O/MMA (v/v) = 6/1, poly(VT-*r-n*BA)-*g*-CNCs/H_2_O = 4 mg mL^−1^	83 400	1.08
3	1	[MMA]_0_ : [CPADB]_0_ : [KPS]_0_ = 400 : 1 : 1, H_2_O/MMA (v/v) = 6/1, poly(VT-*r-n*BA)-*g*-CNCs/H_2_O = 4 mg mL^−1^	84 600	1.11
4	1	[MMA]_0_ : [CPADB]_0_ : [KPS]_0_ = 400 : 1 : 1, H_2_O/MMA (v/v) = 6/1, poly(VT-*r-n*BA)-*g*-CNCs/H_2_O = 4 mg mL^−1^	83 000	1.13
5	1	[MMA]_0_ : [CPADB]_0_ : [KPS]_0_ = 400 : 1 : 1, H_2_O/MMA (v/v) = 6/1, poly(VT-*r-n*BA)-*g*-CNCs/H_2_O = 4 mg mL^−1^	89 400	1.08

## Conclusions

Poly(AN-*r*-BA) was grafted onto CNCs *via* the union of click chemistry and a Mitsunobu reaction for the formation of poly(VT-*r-n*BA)-*g*-CNCs. In this work, poly(VT-*r-n*BA)-*g*-CNCs as a stabilizer, were successfully used to achieve a RAFT-mediated Pickering emulsion polymerization. The polymerization was used for the synthesis of PMMA beads with narrow dispersities and controlled molecular weights under the following conditions: [MMA]_0_ : [KPS]_0_ : [CPADB]_0_ = 400 : 1 : 1, H_2_O : MMA (v/v) = 6 : 1, *T* = 60 °C. Importantly, the poly(VT-*r-n*BA)-*g*-CNCs could be reused and have a broad scope in the development of Pickering emulsions.

## Conflicts of interest

There are no conflicts to declare.

## Supplementary Material

RA-008-C8RA03816C-s001
