# Supplementary material for: RAFT-mediated Pickering emulsion polymerization with cellulose nanocrystals grafted with random copolymer as stabilizer
Source: RSC Adv. 2018 Aug 13;8(50):28660–7. doi: 10.1039/c8ra03816c (PMC9084400; doi:10.1039/c8ra03816c)
Supplement: RA-008-C8RA03816C-s001 [file RA-008-C8RA03816C-s001.pdf]

Electronic Supplementary Material (ESI) for RSC Advances.
This journal is © The Royal Society of Chemistry 2018

Supporting Information

RAFT-mediated Pickering Emulsion Polymerization with Cellulose Nanocrystals Graft Random Copolymer as Stabilizer

Liangjiu Bai[1,2], Xinyan Jiang[1,2], Beifang Liu[1,2], Wenxiang Wang[1,2]*, Hou Chen[1,2]*, Zhongxin Xue[1,2], Yuzhong Niu[1,2], Huawei Yang[1,2], Donglei Wei[1,2]

1. School of Chemistry and Materials Science, Key Laboratory of High Performance and Functional Polymer in the Universities of Shandong Province, Ludong University, Yantai 264025, China.

2. Collaborative Innovation Center of Shandong Province for High Performance Fibers and Their Composites, Ludong University, Yantai 264025, China.

E-mail: wenxiang-tianshi@163.com (W. X. Wang), +Fax: 86-535-6669070; ldupolymchen@163.com (H. Chen), Fax: +86-535-6697933.

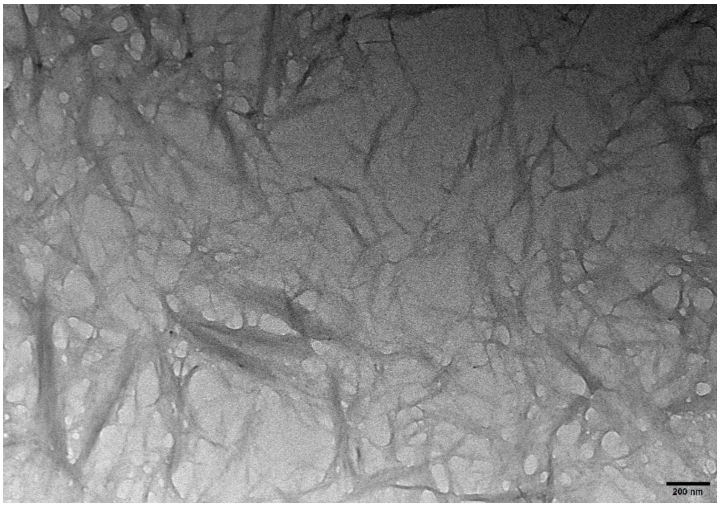

Figure S1. TEM image of CNCs.

Table S1. EDS data of CNCs, Poly(AN-*r*-BA), Poly(VT-*r*-BA) and Poly(VT-*r*-BA)-*g*-CNCs.

Sample	Composition (wt%)			[N]/[O]	[C]/[O]
	C	O	N		
CNCs	50.69	40.31	--	--	1.68
Poly(AN-*r*-BA)	69.70	19.10	11.20	0.67	4.88
Poly(VT-*r*-BA)	62.44	11.30	26.06	2.63	7.37
Poly(VT-*r*-BA)-*g*-CNCs	56.95	20.15	22.90	1.30	3.76

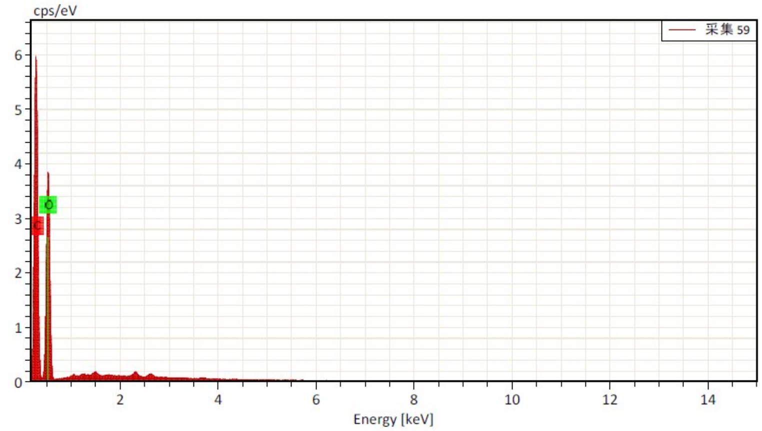
cps/eV
采集 59
O
Energy [keV]

(a)

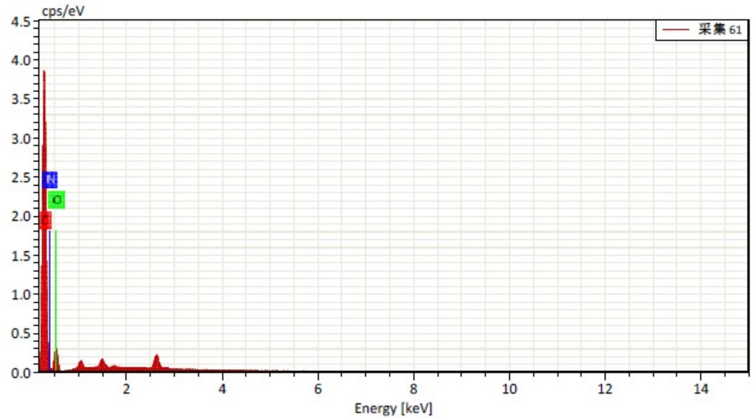
cps/eV
采集 61
N
O
Energy [keV]

(b)

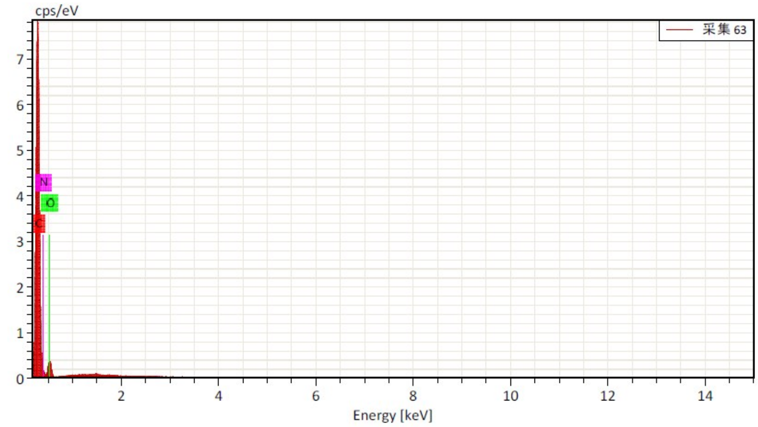
cps/eV
采集 63
N
O
Energy [keV]

(c)

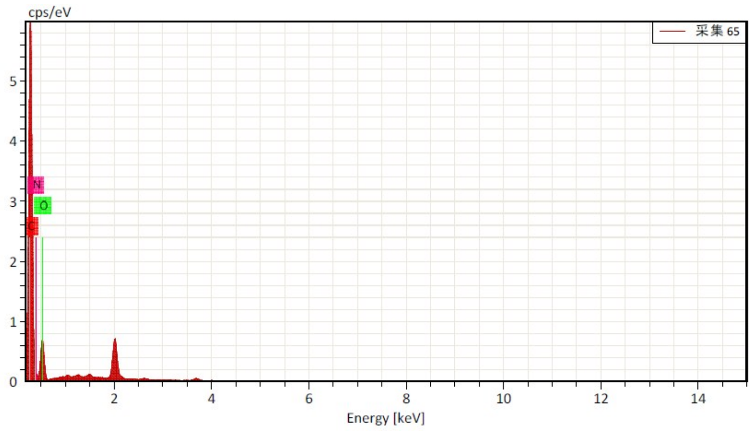

(d)

Figure S2. EDS picture of CNCs (a), Poly(AN-*r*-BA) (b), Poly(VT-*r*-BA) (c) and Poly(VT-*r*-BA)-*g*-CNCs (d).

FT-IR spectra of poly(AN-*r*-BA), poly(VT-*r*-BA), CNCs and poly(VT-*r*-BA)-*g*-CNCs. Poly(AN-*r*-BA), poly(VT-*r*-BA), CNCs and poly(VT-*r*-nBA)-*g*-CNCs was further characterized by IR measurement. The click chemistry of poly(AN-*r*-BA) with sodium azide lead to poly(VT-*co*-BA) containing tetrazolium group. As shown in figure 5, the FTIR spectrums of poly(AN-*r*-BA) and poly(VT-*r*-BA)had their obvious IR characters. In spectrogram line of poly(AN-*r*-BA), the peak absorption at 2240 cm^{-1} was in accordance with C≡N stretching vibration, absent in the IR spectrum of poly(VT-*r*-BA) due to C≡N groups converted into tetrazolium group. Moreover, there was additional peak at 1545 cm^{-1} and 1610 cm^{-1} associated with tetrazolium group, appears with the IR spectrum of poly(VT-*r*-BA). Therefore, these phenomenon manifest successfully preparation of poly(VT-*r*-BA) from poly(AN-*r*-BA). poly(VT-*r*-BA)-CNCs was obtained by Mitsunobu reaction between poly(VT-*r*-BA) and CNCs. In IR spectra of CNCs, all characteristic was found. Meanwhile, the peak at 1723 cm^{-1} attributed to C=O stretching vibration derived from poly(VT-*r*-BA) and 1059 cm^{-1} from CNCs were observed in IR spectrum line poly(VT-*r*-BA)-CNCs at the same time. On the basis of analying these character, it clearly demonstrated that the successful synthesis of poly(VT-*r*-nBA)-*g*-CNCs.

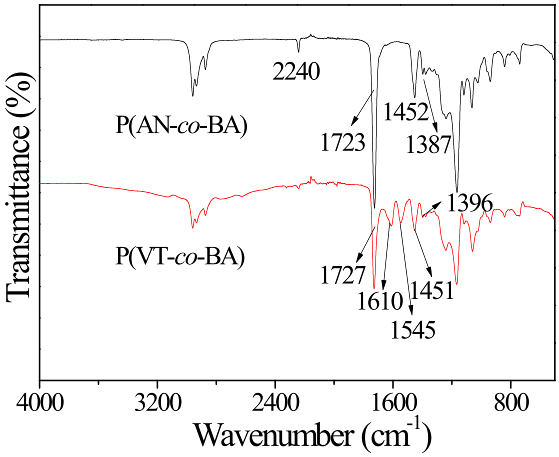

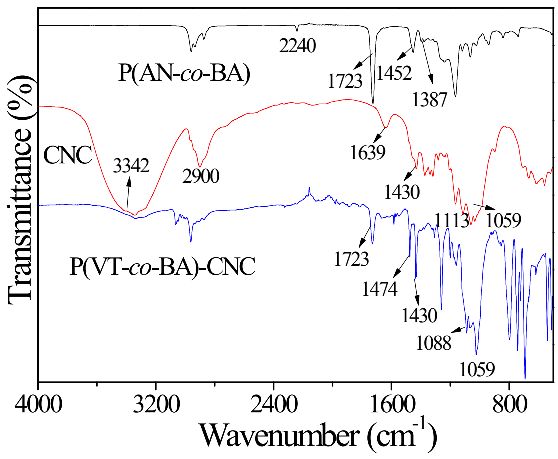

Figure S3. The FT-IR spectrogram of poly(AN-*r*-BA), poly(VT-*r*-BA), CNCs and poly(VT-*r*-BA)-*g*-CNCs.
